# *CORR* Insights^®^: How Do Different Anterior Tibial Tendon Transfer Techniques Influence Forefoot and Hindfoot Motion?

**DOI:** 10.1007/s11999-014-4099-3

**Published:** 2014-12-13

**Authors:** Reggie Charles Hamdy

**Affiliations:** Shriners Hospital for Children, 1529 Cedar Avenue, Montreal, QC H3G 1A6 Canada

## Where Are We Now?

The Ponseti technique is largely accepted today as the preferred and most-commonly used method for the management of idiopathic clubfoot [6, 7]. Although successful in obtaining a satisfactory initial correction, recurrence of deformities is common with this technique, occurring in up to 54% of the feet treated in Ponseti’s original report [6]. Many recurrences happen because of poor compliance with bracing [1]. Dynamic supination of the foot is one of the earliest signs of relapse [3], and is generally caused by weak peronei leading to a muscular imbalance between the invertors and evertors of the foot. If left untreated, this may progress and lead to a stiff deformity. Tibialis anterior tendon transfer (TATT) has been shown to be an effective procedure in restoring muscle balance and correcting this deformity, improve plantar loading, function and satisfaction with low incidence of recurrence [4], yet there is no uniform agreement as to which of the three techniques that have been described—complete transfer through two or three incisions and split transfer—should be used to correct dynamic supination. Furthermore, there is no standardized method to measure the severity of the dynamic supination deformity. Finally, there is also no standard objective method to measure muscular strength in this population of patients, specifically of the peronei. Knutsen and colleagues’ original research on 10 cadavers provide novel findings on the three TATT techniques and recommendations for use depending on the dynamic deformity and weakness of the peronei.

## Where Do We Need To Go?

Perhaps, most importantly, it remains difficult to translate results obtained from adult cadavers to children. Because of this, determining criteria on which the choice of each TATT would be based also remains a challenge.

Although indications for TATT have been reported to include poor contact of the first metatarsal head with the ground while walking or running, weight bearing on the lateral border of the foot, and persistent dorsiflexion of the foot into supination [2], we need to address the important dilemma for anyone who deals with clubfeet: Which of the three transfer techniques of tibialis anterior transfer is indicated and for which cases? The research findings by Knutsen et al. contribute to the current state of knowledge in this area. The authors acknowledge that future clinical trials are required to confirm the indications for each of the tendon transfer techniques in specific clinical presentations. However, before embarking on such clinical trials, we need first to identify a standardized and reproducible measurement of dynamic supination and hence clearly define what is considered a relapse [9]. Objective and reproducible measurements are required to provide scientifically sound findings and cannot be based solely on the surgeon’s appreciation of severity. Second, we also need to identify the best method to quantify the strength of various muscle groups in the foot of a 3- or 4-year-old child, as this may help in the choice of technique. We also need to identify the optimal time frame to perform the tendon transfer following initial correction of the deformity, if the transfer should be performed after the first or second relapse and the role of repeated manipulations and casting in the management of relapses.

## How Do We Get There?

First, in order to address the question of how to measure dynamic supination deformity in a standardized and reproducible manner, a pilot study comparing measurement of dynamic supination using goniometry, pedobarography, and motion analysis techniques is required. Dynamic supination could be measured using goniometry to define the angle from the plantar aspect of the foot in the supine position to the floor or by using pedobarography to measure the orientation of the foot relative to the ground and subsequently the contact area, the contact time and peak pressure during static or dynamic stance [5]. A motion-analysis lab could be useful to determine the foot progression angle during walking using a kinematic model of the foot [8]. Second, testing muscle strength in that age group, should be performed using a reliable and reproducible technique such as hand-held dynamometry [4]. Third, a multisite clinical trial comparing a whole transfer technique using two or three incisions or a split tendon transfer would provide the highest level of evidence in determining the most effective technique in correcting the supination deformity. The choice of tendon transfer technique would be allocated per surgeon, as one technique is typically adopted by a surgeon. When selecting the primary outcome measure in a clinical trial, one needs to keep in mind that the main goal of a TATT is to obtain a plantigrade foot therefore reinforcing the need for a preliminary study to standardize measurement of dynamic supination. A power calculation would be based on the primary outcome. Secondary outcomes would include peroneal muscle power, ROM, position of the foot, and function in order to best represent optimal correction.
